# Ecologic Niche Modeling of *Blastomyces dermatitidis* in Wisconsin

**DOI:** 10.1371/journal.pone.0002034

**Published:** 2008-04-30

**Authors:** Kurt D. Reed, Jennifer K. Meece, John R. Archer, A. Townsend Peterson

**Affiliations:** 1 Marshfield Clinic Research Foundation, Marshfield, Wisconsin, United States of America; 2 Wisconsin Department of Health and Family Services, Madison, Wisconsin, United States of America; 3 Natural History Museum and Biodiversity Research Center, University of Kansas, Lawrence, Kansas, United States of America; University of Sydney, Australia

## Abstract

**Background:**

Blastomycosis is a potentially fatal mycosis that is acquired by inhaling infectious spores of *Blastomyces dermatitidis* present in the environment. The ecology of this pathogen is poorly understood, in part because it has been extremely difficult to identify the niche(s) it occupies based on culture isolation of the organism from environmental samples.

**Methodology/Principal Findings:**

We investigated the ecology of blastomycosis by performing maximum entropy modeling of exposure sites from 156 cases of human and canine blastomycosis to provide a regional-scale perspective of the geographic and ecologic distribution of *B. dermatitidis* in Wisconsin. Based on analysis with climatic, topographic, surface reflectance and other environmental variables, we predicted that ecologic conditions favorable for maintaining the fungus in nature occur predominantly within northern counties and counties along the western shoreline of Lake Michigan. Areas of highest predicted occurrence were often in proximity to waterways, especially in northcentral Wisconsin, where incidence of infection is highest. Ecologic conditions suitable for *B. dermatitidis* are present in urban and rural environments, and may differ at the extremes of distribution of the species in the state.

**Conclusions/Significance:**

Our results provide a framework for a more informed search for specific environmental factors modulating *B. dermatitidis* occurrence and transmission and will be useful for improving public health awareness of relative exposure risks.

## Introduction

Blastomycosis is a potentially fatal infection by the thermally dimorphic fungus *Blastomyces dermatitidis*. The organism exists as a mold at ambient temperatures in the environment, but transforms into a pathogenic yeast form after infectious spores are inhaled by susceptible mammalian hosts [1]. In the United States, most blastomycosis cases occur in the Ohio and Mississippi river valleys, the southeastern states, and around the Great Lakes. Distribution maps published in the medical literature show a broad range, but provide little detail regarding specific habitats or areas posing higher risk of exposure [2]. Clinical experience and occurrence of localized outbreaks suggest that the disease occurs at higher frequencies in a few locations, but is relatively uncommon in other areas of endemicity [3]–[6].

**Figure 1:**
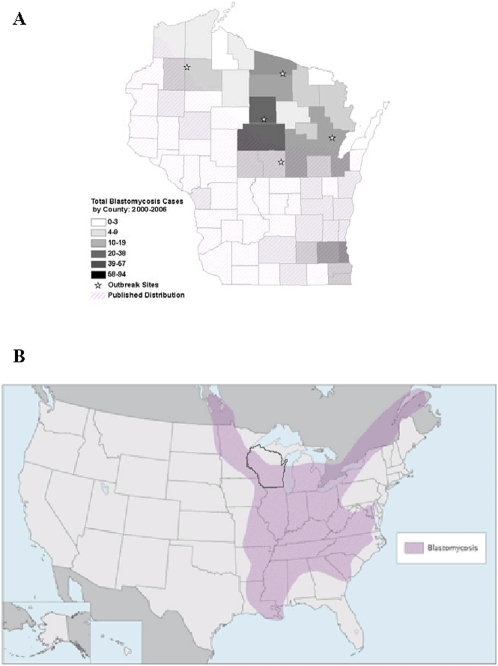
A. Blastomycosis cases reported to the Wisconsin Division of Health and Family Services by county from 2000–2006. Darker shades of gray indicate higher number of cases and lighter shades correspond to fewer cases. Stars are present at sites of reported blastomycosis outbreaks and the hatched area corresponds to the endemic region depicted in the map from Panel B. B. Distribution map of blastomycosis in the United States and Canada. The state of Wisconsin is outlined by the dark line. Note that northcentral Wisconsin is not included as an endemic area in this map published in 2007. Reprinted with permission from the New England Journal of Medicine [9].

In Wisconsin, blastomycosis in humans has been a reportable disease since 1985. Data collected by the Wisconsin Division of Health and Family Services (DHFS) indicate that highest incidences of infection occur in several northcentral counties where mean annual incidence rates range 10.4 to 41.9 per 100,000 (Figure 1A). *B. dermatitidis* is also an important animal pathogen, infecting dogs in Vilas County, also located in the north, at rates estimated as high as 1,420 per 100,000 [7], [8]. In contrast, relatively few cases of blastomycosis are reported from counties in southcentral and southwestern Wisconsin. Despite this information, distribution maps for blastomycosis in the U.S. published as recently as 2007 continue to show northcentral Wisconsin as being non-endemic for the infection (Figure 1B) [2], [9].

Ecologic factors determining presence or absence of *B. dermatitidis* at specific sites are poorly understood, in part because the organism has been recovered from natural settings fewer than two dozen times [4], [10]–[13]. Over the past 50 years, repeated attempts have been made to isolate the fungus from the environment, especially following outbreaks, and efforts have been made to develop better methods of isolation. Methodologies have ranged from animal inoculations to *in vitro* cultivation with selective and non-selective artificial media; more recently, molecular methods have been developed to detect *B. dermatitidis*-specific DNA in environmental samples [14]. Thousands of samples have been tested in areas where occurrence of the disease suggests that the fungus must exist but, in all but a few instances (21 isolates total), negative results have been obtained, so knowledge of the ecologic characteristics and distribution of the fungus continues to be based almost solely on reported disease occurrence.

An unexplored approach for assessing the geographic and ecologic distribution of *B. dermatitidis* is ecologic niche modeling [15]. Previous studies have documented the utility of these models based on disease occurrence locations and multiple environmental variables: e.g., filovirus and monkeypox infections and malaria vectors in Africa [16]–[18], Chagas disease in Mexico [19], and leishmaniasis in South America [20]. The success of these models is enhanced by the ever-increasing availability of comprehensive environmental data generated by earth-orbiting satellites and other remote-sensing platforms. Here, we apply maximum entropy ecologic niche modeling of human and canine cases of blastomycosis to explore the regional-scale ecology and geographic patterns of *B. dermatitidis* occurrence in Wisconsin.

## Methods

### Point-occurrence information

Cases of human and canine blastomycosis in Wisconsin were identified from records of the DHFS and the veterinary division of Marshfield Laboratories (Marshfield, WI). All cases were confirmed by laboratory testing, either by isolation of the agent in culture or by microscopic detection of characteristic yeast forms in biopsy or aspirate specimens. Locations for potential exposure sites in humans were determined from a standardized questionnaire developed and administered by DHFS. For canine cases, a similar questionnaire was developed for completion by veterinarians and pet owners. Although blastomycosis in animals is not a reportable disease in Wisconsin, Marshfield Laboratories is a reference laboratory with statewide coverage for veterinary diagnostic services so we are confident that our coverage area for canine cases is complete. Occurrences with multiple possible exposure sites over broad geographic areas, such as several counties, were excluded from analysis. When reasonable guesses could be made as to the point or general area of exposure (i.e., within 2–3 km), they were included, recognizing that they are likely to be representative of the coarse-scale ecologic conditions. All occurrences were georeferenced to the nearest 0.001°.

### Ecologic niche modeling

Ecologic niches and potential geographic distributions were modeled by the maximum entropy method (Maxent) [21]. Briefly, Maxent is a general-purpose machine-learning method that has been developed and specialized for predicting species' distributions when only presence data are available for analysis. The goal of Maxent is to estimate a probability distribution for species' occurrences by finding the distribution of maximum entropy (i.e., closest to uniform), subject to constraints defined by the environmental features being analyzed [21].

For this study, sample points were represented as pixels with known blastomycosis occurrence. For Maxent modeling it is not necessary to have a complete dataset of all known occurrences, but rather a representative sample that is likely to include most or all of the important habitats. For model development it is better to include only those sample points that are well documented than have a more inclusive dataset which includes gross geocoding errors. Ecologic features included geographic information system coverages representing 64 climate and other environmental variables (see below). The constraints for Maxent modeling are that the expected values for environmental features should match the empirical average for a set of sample points taken from the complete occurrence dataset [22]. The output of the model is a prediction map where each pixel of the study area is evaluated with regard to suitability for *B. dermatitidis* based on how closely the values for environmental features match those of disease occurrence sites. Each pixel is assigned a real-number value ranging from 0 (lowest suitability) to 100 (highest suitability) and displayed on a map.

Coverages summarizing elevation, slope, aspect, flow direction, flow accumulation, and compound topographic index (a measure of the tendency of water to pool) were obtained from the U.S. Geological Survey's Hydro-1K dataset at a resolution of ∼1 km^2^ (URL:http://edc.usgs.gov/products/elevation/gtopo30/gtopo30.html). Nineteen bioclimatic variables derived from monthly temperature and precipitation values were obtained from WorldClim at a resolution of ∼1 km^2^ (URL:http://worldclim.org). These climatic variables include annual values such as mean temperature and precipitation, measures of seasonality (e.g., annual range in temperature and precipitation) and extreme or limiting environmental factors (e.g., temperature of the coldest and warmest month, precipitation of the wettest and driest months). Sixteen-day composites of normalized difference vegetation indices and enhanced vegetation indices derived from the MODIS (or Moderate Resolution Imaging Spectroradiometer) satellites were obtained for the time period 23 April through 2 December, 2002 at a resolution of ∼0.25 km^2^ (URL:http://modis.gsfc.nasa.gov/data/). Additionally, mean values and standard deviations were calculated for each of these MODIS vegetation indices. Coverages summarizing soil characteristics such as soil-carbon density, total nitrogen density, field capacity, wilting point, profile available water capacity, thermal capacity, and bulk density were obtained from the Global Gridded Surfaces of Selected Soil Characteristics dataset at a resolution of ∼100 km^2^ (URL:http://www.daac.ornl.gov). All coverages were re-sampled to 1 km^2^ resolution for analysis, reflecting the approximate precision with which the occurrence data were georeferenced. To explore possible associations between blastomycosis occurrence and waterways, we used a 1∶24,000 hydrography shapefile obtained from the Wisconsin Department of Natural Resources (URL:http://www.dnr.state.wi.us/maps/gis/datahydro.html) and a streams and waterbodies shapefile obtained from the U.S. National Atlas (URL:http://www.nationalatlas.gov/). In order to quantify the association of blastomycosis exposure sites with waterways, we calculated the mean distance of occurrence points from rivers or streams. We also compared those results with mean distances calculated for 100 sets of 156 points chosen randomly from the study area.

All modeling in this study was carried out on a desktop implementation of Maxent (URL:http://www.cs.princeton.edu/schapire/maxent/). The final model was generated using all occurrence points divided randomly into training (50% for model building) and testing (50% for model evaluation) datasets. A jackknife test was used to evaluate individual variable importance in model development, and receiver operating curve analysis was used to assess overall model quality [23]. Predictions were mapped in a geographic information system environment (ArcGIS 9.0, ESRI, Redland, CA).

### Model robustness

To provide a test of the ability of our models to predict the distribution of human or canine blastomycosis in areas from which no input data are present, we used a spatially stratified subsetting procedure (see references [17] and [20] for other examples). Models were generated with human-only cases, canine-only cases, random and checkerboard-stratified subsets of occurrences and northern or southern locations of occurrence points. Model quality, based on the independent testing datasets reserved prior to modeling, was evaluated with one-tailed binomial probability distributions. We compared those results with those expected if the model was generated from a random distribution of blastomycosis occurrence points.

## Results

One hundred forty-five cases of human blastomycosis were identified from DHFS records for the period from 1 January 2005 through 31 May 2006. Information sufficient for geocoding of potential exposure locations was available for 103 cases. Cases excluded involved 11 with missing or incomplete questionnaires, 30 with poor documentation of the clinical diagnosis or insufficient information to allow a reasonable estimate of the exposure site, and one that fell outside the boundaries of the environmental dataset. Twenty-one cases were associated with a blastomycosis outbreak in northcentral Wisconsin that lasted from December 2005 through April 2006. Seventy-five canine cases of blastomycosis were identified through laboratory records of which information was sufficient for georeferencing in 53 cases; 51 of these cases occurred in 2005–2006 and the remaining 2 in 2003–2004.

**Figure 2:**
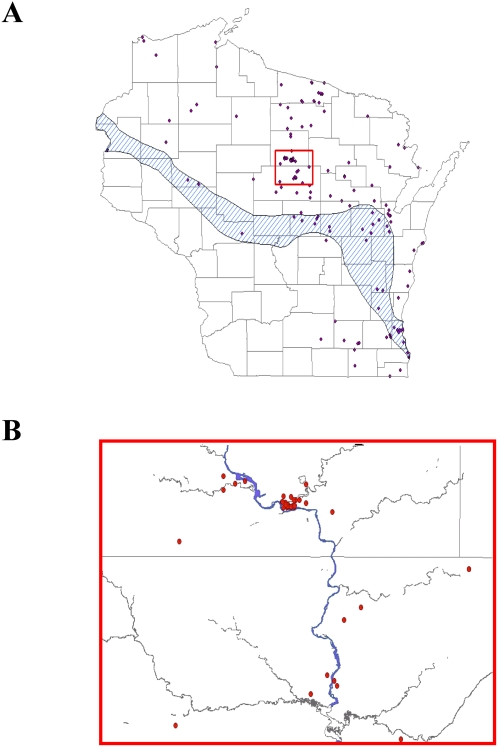
A. Summary of known occurrences of blastomycosis in Wisconsin. The cross-hatched area represents the “tension zone,” an ecological gradient of climate change that separates the northern and southern climatic zones of Wisconsin (adapted from reference [32]). Over 90% of blastomycosis cases occurred within the tension zone or in the northern climatic zone. The area outlined by the red box is shown in greater detail in 2B. B) Association of blastomycosis cases with the Wisconsin River (blue line) and its tributaries (gray lines) in northcentral Wisconsin. Straight lines are county boundaries. The dense cluster of cases at the top of the figure corresponds to the location of a community outbreak that occurred in late 2005 and early 2006.

Exploratory spatial analysis revealed that exposure sites were distributed broadly across northern Wisconsin, and in a narrow band along the Lake Michigan shoreline further south. In general, this pattern coincides with the northern climate zone of Wisconsin, where high frequencies of cool, dry, Arctic air from Canada dominate weather patterns and produce relatively long winters with abundant snow and cool temperatures. In contrast, the southern climate zone tends to be dominated by air from the Pacific Ocean and the Gulf of Mexico, which converges to produce warmer winters with less snow and longer summers with more rainfall. Curtis' tension zone refers to a diagonal band across the state that divides the two climate zones and encompasses areas with features of both the northern and southern climates. Of 156 blastomycosis exposure locations, 146 (94%) were within the tension zone or the northern climate zone (Figure 2A).

To determine whether blastomycosis exposure locations were associated with waterways, occurrence points were overlaid on the 1∶24,000 hydrography dataset. In northcentral Wisconsin, the vast majority of blastomycosis occurrences were in close proximity to the Wisconsin River and its major tributaries (Figure 2B). Similarly, in eastcentral Wisconsin, cases were clustered along the Fox River and tributaries (not shown). The 156 blastomycosis occurrence points averaged 2.7 km from rivers and other waterbodies. In contrast, the average distance to water was 4.1±0.24 km for the 100 sets of random points, and the 2.7 km we observed was a shorter distance than any of the randomized replicates (P<0.01).

**Figure 3:**
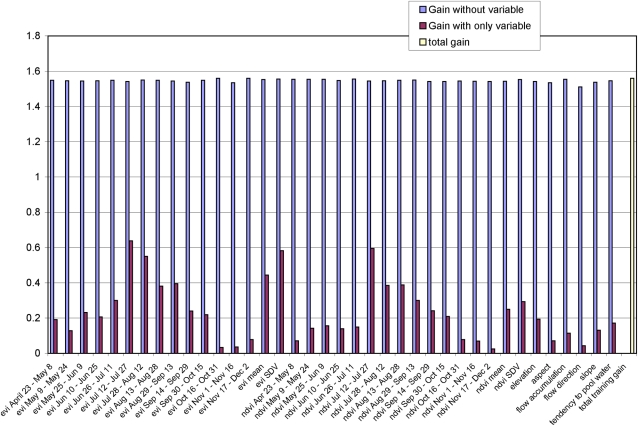
Jackknife test of individual variable importance in the development of the final Maxent model relative to overall model quality or “total gain” (yellow bar). For each environmental variable the blue bar shows how much the total gain is diminished if that specific variable is excluded from analysis. In contrast the red bar shows the gain achieved if a single variable is used alone and the remaining variables are excluded from analysis. evi = enhanced vegetation index; ndvi = normalized difference vegetation index; SDV = standard deviation. The date range associated with each vegetation index corresponds to the specific 16-day interval over which the composite value was collected.

The importance of individual environmental variables in developing the prediction map was assessed by generating an initial model incorporating all blastomycosis occurrence locations and the entire suite of environmental variables. Jackknife analysis showed that bioclimatic variables and soil characteristics made only small contributions to model development when used alone or when each coverage was excluded individually (data not shown). Thus, those variables were omitted in subsequent models. In contrast, environmental variables contributing the most to model development when used in isolation were enhanced vegetation indices and normalized difference vegetation index composites from late June through mid September along with the mean and standard deviation of the enhanced vegetation indices (Figure 3). The environmental variable influencing the model most when omitted (i.e., that had the most information not present in the other variables) was flow direction, a topographic data layer summarizing the direction of water flow from each cell of the digital elevation model to its steepest down-slope neighbor.

**Figure 4:**
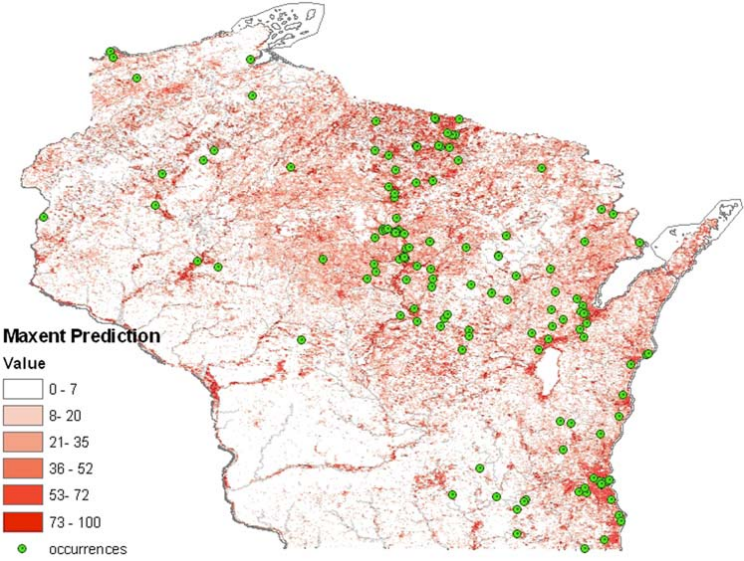
Predicted potential geography of *B. dermatitidis* in Wisconsin based on maximum entropy modeling using all occurrence points. Low values (white and light red shades) represent habitats that have low suitability to support the fungus. Darker shades of red indicate favorable habitats, and are clustered around waterways in northern Wisconsin and along the Lake Michigan shoreline in the south.

Our model based on vegetation indices and topographic variables was consistent with our exploratory analysis (Figure 4). Favorable habitat was concentrated within or above Curtis' tension zone and along waterways. However, within the tension zone and northern climatic zones, considerable variation in prediction of occurrences was also observed. In general, areas of highest predicted occurrence were in landscapes characterized by moist soil and numerous waterways, whereas lower prediction values were observed in landscapes with drier, well-drained soils. Based on receiver operating curve analysis, our model performed very well with a calculated area under the curve of 0.939 compared to an expected value of 0.5 if the prediction were to be random (P<0.001).

**Figure 5:**
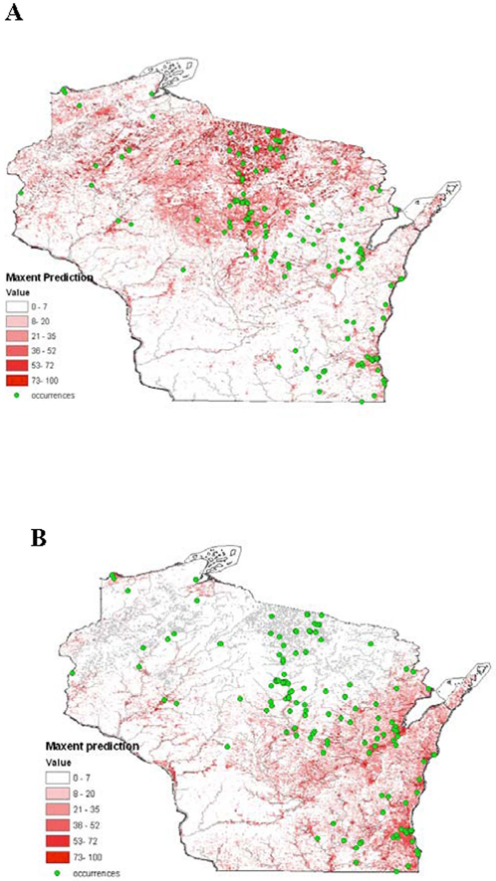
Geographic projection of predicted potential distribution of blastomycosis in Wisconsin based on northern (Panel A) and southern (Panel B) occurrence locations only.

Subsetting analyses, in which a portion of the occurrence data was used to train models that were then tested with the rest of the data, allowed us to further examine the generality and predictive ability of the niche models. We found that models generated with human-only and canine-only cases predicted each other well and that random or checkerboard subsets of occurrences predicted well also (binomial probabilities, all P<0.001). However, the large geographic area and multiple landscapes comprising this regional-scale analysis, as well as the fact that Wisconsin covers only part of the range of the species in question, make it likely that different factors could modulate presence or absence at the extremes of the distribution of the species. To investigate this possibility, we compared models generated with all occurrence points with those derived from points limited to the northern or southern halves of the study area. The map generated from the northern points included areas of high predicted occurrence in densely populated areas of southeastern Wisconsin, suggesting that environmental factors important for sustaining *B. dermatitidis* in that region are similar to those in the northern forests (Figure 5A). In contrast, the model generated based on southern Wisconsin occurrence points was much less successful in predicting northern occurrence areas (Figure 5B).

## Discussion

Ecologic niche modeling and distribution prediction with Maxent, the Genetic Algorithm for Rule-Set Prediction and other methods are being used increasingly to investigate how environmental factors might influence infectious disease transmission [15]. These techniques have been found to be particularly useful in cases where only presence data are available and other more general-purpose statistical methods, such as multivariate logistic regression, are not applicable [24]–[26]. The geographic predictions generated by these methods are valuable because they represent powerful hypothesis-generating tools [27]. For infectious disease research, maps are particularly useful for displaying disease risks for human and animal populations, providing clues as to specific biotic and abiotic factors that modulate pathogens in natural settings, and determining potential reservoir hosts for zoonotic agents.

Historically, blastomycosis ecology has been difficult to investigate for several reasons. Unlike the systemic fungal pathogens *Histoplasma capsulatum* and *Coccidiodes immitis*, no reliable and readily available skin tests or methods of soil isolation exist for blastomycosis to help outline its geographic distribution. Additionally, lag times between exposure to infectious spores and development of clinical symptoms can be highly variable for blastomycosis, ranging from several weeks to several months [28], which can result in patients not being able to recall accurately all activities that could have resulted in exposure. We attempted to minimize this problem by excluding cases in which extensive travel occurred in months prior to diagnosis, but accuracy of geocoding is clearly a limitation of our study. Another limitation is that we did not control for prolonged climate variations (e.g., drought or heavy rainfall) that could be highly relevant to blastomycosis transmission dynamics. For example, Proctor and colleagues suggested that multiple high-risk environmental foci for blastomycosis may have developed in northern Wisconsin following a 5-year period of diminished precipitation [29].

Our analysis showed that blastomycosis occurrence points were significantly closer to rivers and lakes than replicate sets of points chosen randomly. This association with waterways could be driven, at least in part, by the fact that we included a significant number of cases (n = 21) that were part of an outbreak occurring in a small community along the Wisconsin River. However, our findings are very consistent with previous studies of blastomycosis in Wisconsin that document strong associations between exposure locations and proximity to waterways [4], [6], [30]. For example, epidemiologic analysis identified specific activities that were considered risk factors for infection: visiting and playing on a beaver dam [4], fishing and climbing along river banks [29], and canoeing [6]. Here, we observed clustering of blastomycosis cases along rivers in northcentral and eastcentral Wisconsin. We did not have sufficient numbers of cases to make a visual assessment of the association between waterways and blastomycosis in densely populated southeastern Wisconsin. Baumgardner and colleagues recently analyzed blastomycosis cases in Milwaukee, Wisconsin, with most cases being associated with open urban watersheds [31].

Maximum entropy modeling provided a broad-extent view of *B. dermatitidis* distributions at higher resolution than maps generated in the past from public health data aggregated at the county level [7]. In general, blastomycosis appears to be a disease of the tension zone and northern climatic zone in Wisconsin. The concept of an ecological tension zone was first described by J. T. Curtis in 1959 as part of his seminal work on geographic distributions of plant species [32]. He noted that the two distinct climatic regions of Wisconsin strongly influenced the distribution of certain plant species. Although it seems likely that similar factors could modulate distributions of molds in natural settings, we recognize that climate is an extremely complex phenomenon and that dividing Wisconsin into two climate zones is an oversimplification. Our model bears this out by demonstrating significant heterogeneity of prediction values within the northern climate and tension zones, as well as several areas with high prediction values in the southern zone. Additionally, jackknife analysis did not identify any specific climate variable that contributed significantly to our model development. This suggests that, although the tension zone may represent a valid ecologic concept, the underlying environmental determinants remain poorly understood.

The ecological landscapes of Wisconsin are complex, with at least 16 distinct landscape types currently recognized [33]. In addition to climate, numerous other biotic and abiotic factors contribute to the formation of these habitats and, consequently, hold potential for influencing the presence or absence of *B. dermatitidis*. In our study, jackknife analysis revealed that numerous environmental variables made at least a small contribution to overall model development and that composite vegetation indices from late June through mid-September had the largest impact. This time period coincides with peak vegetation in many areas of Wisconsin. Exclusion of the variable associated with water flow direction had the largest impact on model development when excluded from analysis. This finding probably relates, at least indirectly, to the importance of waterways reported by other investigators and observed in our exploratory spatial analysis.

A significant finding of this study was that, while finer-scale subsetting exercises resulted in models with significant predictive ability, a model generated with occurrence points restricted to the southern half of the state in large part failed to predict known occurrence areas in the north. Although other explanations are possible (e.g., small sample sizes), this result suggests that different environmental factors may be important at the extremes of the study area. This point is particularly relevant given that our analyses did not cover the full geographic distribution of the species, and as such may not summarize the full ecological range. Baumgardner and colleagues suggested recently that the relative paucity of inland waterways and lack of sand soils in Milwaukee County explains, at least in part, the significantly lower incidence of blastomycosis there compared to the Eagle River area of northcentral Wisconsin [31]. Our analysis did not reveal soil characteristics to be important in model development. However, our soil characteristics dataset was at a very coarse scale (∼100 km^2^ resolution) and may not have included variables that are highly important for sustaining *B. dermatitidis* in nature. Another possibility yet to be explored is that different strains of *B. dermatitidis* exist that are better adapted to certain environments and that may vary in their pathogenicity for humans. This question is a subject of ongoing investigations.

Further refinements of our model will be possible as high resolution soil and other environmental datasets become available in the future. Additionally, model validation may be feasible by using molecular methods to detect *B. dermatitidis*-specific DNA in environmental samples. In the interim, the public health response to the problem of blastomycosis in Wisconsin remains one of early detection and treatment of clinical illness and reporting of human cases for epidemiologic purposes. The results of this study should help in these efforts by providing patients and health care providers with a more detailed and realistic view of potential high risk areas for blastomycosis exposure. Additionally, if a safe and effective vaccine were made available, the results of this investigation could help guide patient selection for prophylaxis.
